# Sphingosine-1-phosphate promotes ovarian cancer cell proliferation by disrupting Hippo signaling

**DOI:** 10.18632/oncotarget.15677

**Published:** 2017-02-24

**Authors:** Qianlan Fan, Yuan Cheng, Hsun-Ming Chang, Masashi Deguchi, Aaron J. Hsueh, Peter C.K. Leung

**Affiliations:** ^1^ Department of Obstetrics and Gynaecology, British Columbia Children's Hospital Research Institute, University of British Columbia, Vancouver, British Columbia, V5Z 4H4, Canada; ^2^ Department of Obstetrics and Gynecology, Stanford University School of Medicine, Stanford, CA 94305-5317, USA

**Keywords:** Hippo, YAP, CCN1, CCN2, ovarian cancer

## Abstract

Epithelial ovarian carcinomas account for more than 90% of human ovarian cancers and have become the primary cause of death for gynecological malignancies. Unlimited cell proliferation and resistance to cell apoptosis contribute to the development of ovarian cancers. However, the underlying mechanisms involved in these processes in epithelial ovarian carcinomas are yet poorly understood. In the present study, we examined the Hippo signaling gene expression and investigated the effects of Sphingosine 1-phosphate (S1P) on cell proliferation and the underlying mechanisms in human ovarian cancer cell lines, OVCAR3 and SKOV3. Our results demonstrate that S1P disrupts Hippo signaling by reducing YAP phosphorylation and increasing the expression of CCN1 and CCN2 in both ovarian cancer cells. Furthermore, the increase in CCN1/CCN2 expression contributes to the S1P-induced increase in cancer cell proliferation.

## INTRODUCTION

Epithelial ovarian carcinomas (EOC) account for more than 90% of human ovarian cancers, which have become the primary cause of death for gynecological malignancies in the western countries [1]. Lacking highly sensitive and specific methods for early detection, a majority of patients with the initial diagnosis of EOC are at advanced stages (FIGO classification stage III and stage IV) [1]. Despite aggressive therapy, the clinical outcomes in advanced EOC remain comparatively poor [1]. Therefore, finding a reliable method for early diagnosis of this malignant disease is urgently required. Two major processes, unlimited cell proliferation and resistance to cell apoptosis, contribute to the development of ovarian cancers. However, the underlying mechanisms involved in these processes in EOC are yet poorly understood.

Sphingosine 1-phosphate (S1P) is a potent bioactive sphingolipid metabolite that is essential for maturation of blood vessels in the mammalian development process [2]. Outside the endothelial system, S1P has been shown to modulate various cell functions, including proliferation, migration, invasion, adhesion and angiogenesis, which are critically involved in the development of many malignancies [2–4]. Studies have shown that S1P is aberrantly expressed in ovarian cancer cells, and S1P level is significantly increased in the ascites fluid from ovarian cancer patients [5, 6]. Notably, clinical data have demonstrated that elevated S1P level is associated with poor prognosis in ovarian cancer patients [6]. Moreover, sphingosine kinase 1 (SPHK1), a key enzyme for the conversion of sphingosine to S1P, is overexpressed in ovarian cancer tissues and cultured ovarian cancer lines [7]. Taken together, emerging evidence suggests that S1P may become a diagnostic and prognostic biomarker for ovarian cancers.

Hippo signaling pathway is evolutionarily conserved in metazoan animals that control organ size and tumorigenesis through the regulation of cellular proliferation and survival [8, 9]. This developmental signaling consists of several negative growth regulators acting in a kinase cascade that ultimately phosphorylate and inactivate key transcriptional co-activators, Yes-associated protein (YAP) and PDZ-binding motif (TAZ) [10]. In mammals, the core components of the Hippo signaling kinases include Mst1/2, SAV1, Lats1/2, Mob1a and Mob1b [11]. In normal cells, Hippo signaling restrains cells from proliferation by inhibiting YAP/TAZ activities [12]. When Hippo signaling is disrupted, decreases in YAP phosphorylation increase nuclear levels of YAP, and subsequently nuclear YAP and TAZ act in concert with downstream transcriptional enhancer factor TEF-1 (TEAD) transcriptional factors to increase the expression of downstream growth factors [13]. Indeed, the dysregulation of Hippo pathway has been associated with diverse pathological conditions, including developmental defects, tissue overgrowth and cancers [14]. In ovarian cancer studies, the Hippo effector YAP has been found as an oncogene that is overexpressed in epithelial ovarian cancers [15]. Also, the co-expression of YAP and TEAD4 has been determined as a prognostic marker for poor ovarian cancer survival [16].

The CCN proteins, a set of cysteine-rich regulatory proteins, contain six family members, namely CCN1 to CCN6 and are induced by various growth factors, cytokines, or cellular stress [17]. These proteins are important signaling mediators in stem cell differentiation and tumorigenesis [17]. CCN1 and CCN2 are aberrantly expressed in many types of cancer tissues and are involved in the processes of angiogenesis and tumorigenesis [18]. In particular, CCN1 and CCN2 are the downstream targets of YAP/TAZ and TEAD transcription factors to modulate the expression of multiple genes related to cell proliferation and apoptosis, in response to mechanotransduction [10]. Given the important roles of S1P and Hippo signaling pathway in the tumorigenesis of ovarian cancer, we sought to investigate the effects of S1P on YAP and its downstream effectors CCN1 and CCN2 genes. The aim of this study was to examine the effects of S1P on cell proliferation and the underlying mechanisms in human ovarian cancer cells.

## RESULTS

### S1P induces a decrease in phosphorylated YAP and a nucleus translocation of YAP in OVCAR3 and SKOV3 cells

**Figure 1:**
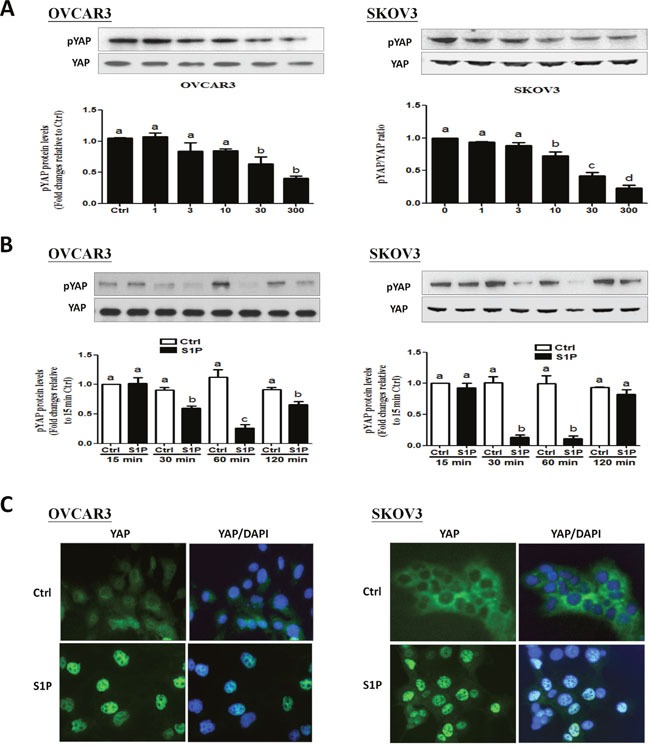
S1P induces a decrease in phosphorylated YAP and a nucleus translocation of YAP in OVCAR3 and SKOV3 cells **(A-B)** OVCAR3 or SKOV3 cells were starved in serum-free medium for 12 h and then stimulated with **(A)** different concentrations of S1P for 30 min or with **(B)** 300 nM S1P for the indicated times, the protein levels of phosphorylated YAP were measured using Western blot analysis. **(C)** OVCAR3 or SKOV3 cells were starved in serum-free medium for 12 h and then stimulated with 300 nM S1P for 30 min, the YAP subcellular localization (green) or DAPI nucleus localization (blue) was determined using immunofluorescence staining. The results are expressed as the mean ± SEM from at least three independent experiments. All samples were compared using one-way ANOVA followed by Tukey's multiple comparison tests, and values without a common letter (a, b, c and d) are significantly different (*P*<0.05).

The transcription co-activator YAP is an important downstream effector of the Hippo pathway, which can regulate tumorigenesis in various tumors, including breast cancer, ovarian cancer, and hepatocellular carcinomas [19]. We first investigated the effects of S1P on YAP phosphorylation in OVCAR3 and SKOV3 cells. Following S1P treatment, the protein levels of phosphorylated YAP (pYAP) and total YAP (YAP) were measured using Western blot analysis. Our results showed that treatment with S1P induced a rapid YAP dephosphorylation at S127 site in a time- and dose-dependent manner (Figure 1A and 1B). In addition, the immunofluorescent staining showed that YAP was initially localized in the cytoplasm. However, YAP proteins were translocated into the nucleus 30 min after S1P treatment (Figure 1C).

### S1P up-regulates the expression of CCN1 and CCN2 in OVCAR3 and SKOV3 cells

**Figure 2:**
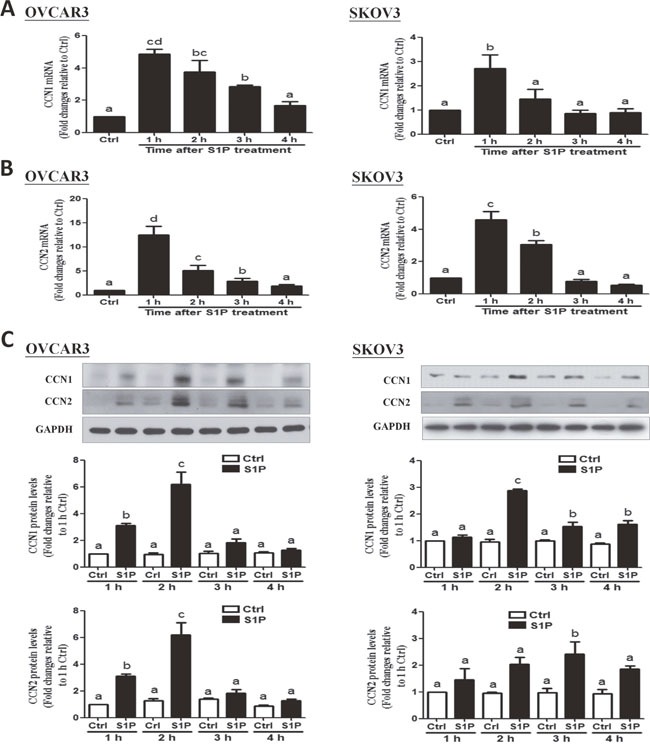
S1P up-regulates the expression of CCN1 and CCN2 in OVCAR3 and SKOV3 cells **(A)** Cells were treated with vehicle control (Ctrl) or 300 nM of S1P for 1, 2, 3 and 4 h, and the mRNA levels of CCN1 **(A)** and CCN2 **(B)** were analyzed using RT-qPCR. **(C)** Cells were treated with 300 nM of S1P for 1, 2, 3 or 4 h, and the protein levels of CCN1and CCN2 were analyzed using Western blot analysis. The results are expressed as the mean ± SEM from at least three independent experiments. All samples were compared using one-way ANOVA followed by Tukey's multiple comparison tests, and values without a common letter (a, b, c and d) are significantly different (*P*<0.05).

To investigate to effects of S1P on the expression of the downstream growth factors, CCN1 and CCN2, we treated the cells (OVCAR3 and SKOV3) with vehicle control or 300 nM of S1P for different time points (1, 2, 3 or 4 h). The results showed that S1P treatment promptly increased the mRNA levels of CCN1 (Figure 2A) and CCN2 (Figure 2B), with the maximal effects happened at 1 h after treatment. Consistent with the results from mRNA, the Western blot analysis revealed that S1P significantly increased the protein levels of CCN1 and CCN2, with the maximal effects happened at 2 h or 3 h after treatment (Figure 2C).

### YAP is involved in the S1P-induced up-regulation of CCN1 and CCN2 in OVCAR3 and SKOV3 cells

**Figure 3:**
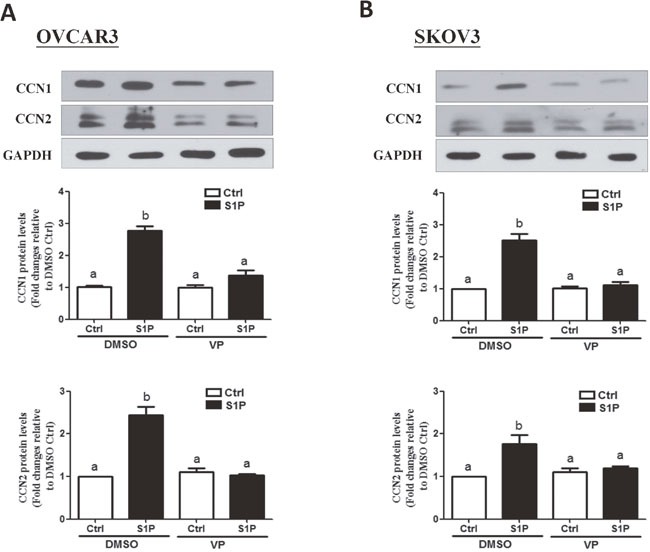
Verteporfin (VP) abolishes the S1P-induced increases in protein levels of CCN2 and CCN1 in OVCAR3 and SKOV3 cells **(A-B)** Cells were pre-treated with 10 μM of VP for 10 min, and then were added with 300 nM of S1P for additional 2 h. The protein levels of CCN1 and CCN2 were analyzed using Western blot analysis. The results are expressed as the mean ± SEM from at least three independent experiments. All samples were compared using one-way ANOVA followed by Tukey's multiple comparison tests, and values without a common letter (a, b, c and d) are significantly different (*P*<0.05).

Next, we determined the functional role of YAP in the S1P-induced up-regulation of CCN1/CCN2. Recent drug library screening have identified a small molecule inhibitor verteporfin (VP), which is capable of inhibiting the association of YAP with the downstream TEAD factors, leading to the suppression of YAP-induced cellular functions [19]. To determine the function of YAP in S1P induced gene regulation, we used VP to block YAP and TEAD complex formation. As shown in Figure 3A and 3B, pretreatment of the cells (OVCAR3 and SKOV3) with 10 μM of VP for 10 min totally abolished the S1P-induced increases in the protein levels of CCN1 and CCN2.

### S1P promotes cell proliferation in OVCAR3 and SKOV3 cells

**Figure 4:**
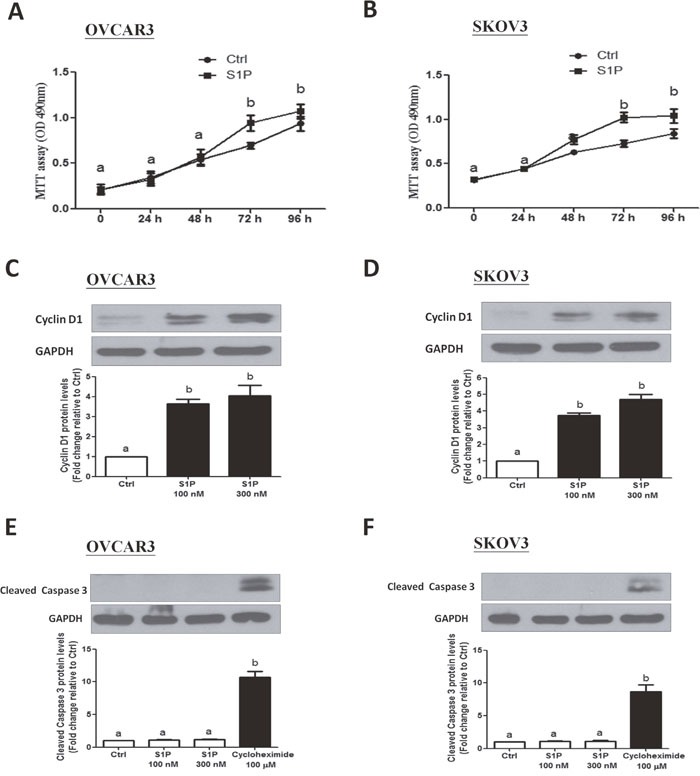
S1P stimulates cell proliferation in OVCAR3 and SKOV3 cells **(A-B)** Cells were seeded in a 96-well plate. After incubation for 24 h, S1P or PET solution (vehicle control) were added for additional 72 h. The cell viability was evaluated using an MTT assay. **(C-D)** Cells were treated with 100 or 300 nM of S1P for 24 h, and cyclin D1 protein levels were examined using Western blot analysis. **(E** and **F)** Cells were treated with S1P (100 or 300 nM) or cycloheximide (100 μM) for 24 h, and the cleaved caspase 3 protein levels were examined using Western blot analysis. The results are expressed as the mean ± SEM from at least three independent experiments. All samples were compared using one-way ANOVA followed by Tukey's multiple comparison tests, and values without a common letter (a, b, c and d) are significantly different (*P*<0.05).

Since S1P is a potent mitogen, we next investigated whether S1P can promote cell proliferation in human ovarian cancer cells. Using a proliferation assay, our results showed that treatment of the cells (OVCAR3 and SKOV3) with 300 nM of S1P for 72 h significantly increased the cell viability (Figure 4A and 4B).

In most somatic cells, cell proliferation is regulated by the cell cycle. To determine whether cell cycle was a contributing factor to cell growth promotion, we investigated the involvement of cyclin D1 (a member of the cyclin protein family) in the S1P-induced increase in ovarian cancer cell proliferation. As shown in Figure 4C and 4D, treatment with S1P (100 or 300 nM) for 24 h significantly increased the protein levels of cyclin D1 in both OVCAR3 (Figure 4C) and SKOV3 (Figure 4D) cells. Since cell number is dependent not only on cell proliferation, but also on cell death, we further conducted experiments to rule out the possibility that apoptosis in ovarian cancer cells could be triggered by S1P. Compared to treatment with 100 μM cycloheximide (a potent inducer of cell apoptosis), treatment of OVCAR3 (Figure 4E) or SKOV3 (Figure 4F) cells with S1P (100 or 300 nM) for 24 h did not influence the cleaved caspase 3 levels examined using Western blot analysis.

### CCN1 and CCN2 mediate the S1P induced cell proliferation in OVCAR3 and SKOV3 cells

**Figure 5:**
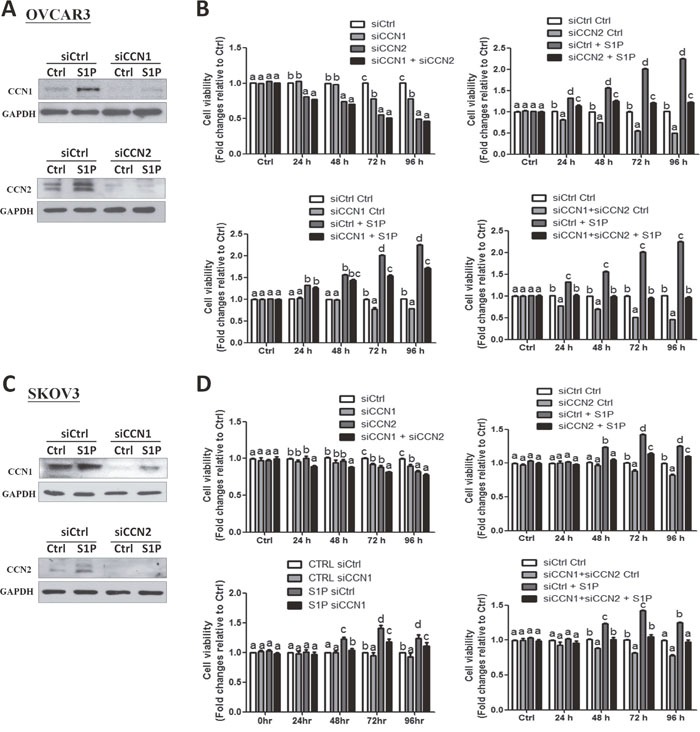
CCN1 and CCN2 mediate the S1P-induced cell proliferation in OVCAR3 and SKOV3 cells **(A** and **C)** OVCAR3 **(A)** or SKOV3 **(C)** cells were transfected for 48 h with 50 nM control siRNA (siCtrl), CCN1 siRNA (siCCN1) or CCN2 siRNA (siCCN2), and then treated with S1P for additional 2 h. The protein levels of CCN1 and CCN2 were analyzed using Western blot analysis. **(B** and **D)** OVCAR3 **(B)** or SKOV3 **(D)** cells were transfected for 48 h with 50 nM siCtrl, siCCN1, siCCN2 or combined siCCN1 and siCCN2 (siCCN1+siCCN2), and then treated with S1P for 0, 24, 48, 72 or 96 h. The cell viability was analyzed using an MTT assay. The results are expressed as the mean ± SEM from at least three independent experiments. All samples were compared using one-way ANOVA followed by Tukey's multiple comparison tests, and values without a common letter (a, b, c and d) are significantly different (*P*<0.05).

To date, the fundamental role of CCN1 and CCN2 in the development of ovarian cancers remains unclear. To further investigate the roles of CCN1 and CCN2 in the S1P-induced cell proliferation, we used small interfering RNA targeted knock down approach in two ovarian cancer cell lines. Using Western blot analysis, we confirmed that transfection with 50 nM CCN1 siRNA or CCN2 siRNA for 48 h significantly down-regulated the protein levels of CCN1 or CCN2 compared with the transfection with non-targeting control siRNA (Figure 5A and 5C). Notably, compared with the control siRNA, knockdown of either CCN1 or CCN2 for 72 h increased the cell proliferation in both OVCAR3 and SKOV3 cells, and these stimulatory effects were further enhanced by concomitant knockdown of both CCN1 and CCN2 (Figure 5B and 5D). In addition, knockdown of either CCN1 or CCN2 attenuated the S1P-induced increases in cell proliferation (Figure 5B and 5D). Furthermore, concomitant knockdown of CCN1 and CCN2 (siCCN1 + siCCN2) completely abolished the S1P-induced cell proliferation in both cell lines (right lower panels of Figures 5B and 5D). These results indicate that both CCN1 and CCN2 are required for the S1P-induced cancer cell proliferation.

### Reduced SPHK1&2 mRNA levels are associated with the increased month disease free rates and the increased month overall survival rates in ovarian serous cystadenocarcinomas

Using the Kaplan-Meier analysis, data from The Cancer Genome Atlas (TCGA) of 607 patients with serous ovarian cystadenomas showed that the reduced SPHK1&2 mRNA levels (lower than the two-thirds) are associated with increased month disease free rates and increased month overall survival rates in this cancer type (Supplementary Figures 1B and 1C). Furthermore, there is a trend towards the reduced mRNA expression levels of CCN1 and CCN2 associated with the reduced SPHK1&2 mRNA levels (Supplementary Figures 1D and 1E).

### Reduced CCN1/CCN2 mRNA levels are associated with the elevated disease free survival rates and the elevated overall survival rates in ovarian serous cystadenocarcinomas

The cBioPortal for Cancer Genomics from The Cancer Genome Atlas was used to query 607 patients with serous ovarian cystadenocarcinomas. As shown in Supplementary Figure 2, reduced CCN1/CCN2 mRNA levels (lower than the two-thirds) were associated with higher month disease free rates (Supplementary Figure 2B) and higher month overall survival rates (Supplementary Figure 2C) displayed as the Kaplan-Meier survival curves.

## DISCUSSION

To enhance the molecular understanding of carcinogenesis and diversity of ovarian cancers, a comprehensive expression analysis of cancer-related signaling networks is urgently required prior to development of individual therapy strategies. In the present study, we examined the Hippo signaling gene expression levels and found that all of the genes examined are expressed in two EOC cell lines, even though two cell lines have the different genetic background (data not shown). In normal physiology process, Hippo signaling pathway negatively regulates liver growth and is a suppressor of liver tumor formation. In mice, specific deletion of SAV1 or Mst1/2 genes in hepatocytes leads to the phenotype of enlarged livers [20, 21]. Likewise, Hippo signaling controls heart size by restricting cardiomyocyte proliferation, and conditional deletion of SAV1 may lead to enlarged hearts [22]. Hippo signaling is also critical for tissue regeneration and the expansion of stem cells and tissue-specific progenitor cells [23]. Our studies have demonstrated that all of the key components of Hippo signaling are expressed in two cancer cell lines. However, the expression pattern is different. Comparing with the normal ovarian surface epithelium cells OSE364 cells, FMD6 expression levels are relatively lower, whereas the SAV1 expression levels are higher in EOC cells (data not shown). These results indicate that Hippo signaling activity varies between normal ovarian epithelium cells and ovarian cancer cells.

The retinoblastoma protein (Rb) and the p53 transcription factor are two of the most prominent regulators that are disrupted in cancer cells because these two interconnecting signaling pathways modulate the cell proliferation and apoptosis processes [24]. The SKOV3 cells were derived from an ovarian cancer patient without any mutation or deletion of Rb and p53, whereas OVCAR3 cells contain a mutant p53 at R248Q position [25–27]. In fact, about half of all of the cancers appear to harbor a mutation in p53 [28]. Our results are showing that treatment with S1P induced cell proliferation in both EOC cells to indicate that this effect can be mediated by mechanisms that are independent of p53/Rb. Indeed, we demonstrated for the first time that S1P promotes cell proliferation by inhibiting YAP phosphorylation and increasing intranucleus YAP activity, and subsequently triggers the downstream CCN growth factor expression. Consistent with our results, it is well documented that S1P is an oncogenic factor that is frequently deregulated in various cancers [4]. Apart from S1P, the S1P receptors, S1P-metabolizing enzymes and sphingosine kinase (SK) have been demonstrated to be aberrantly expressed in various cancers, such as gastric cancer, colon cancer, breast cancer and glioblastoma [4]. Similarly, a previous study has revealed that S1P could stimulate cancer cell migration and invasion in OVCAR3 cells [29]. Notably, Kaplan-Meier analysis of serous ovarian cystadenomas from The Cancer Genome Atlas (TCGA) (n=607) shows that samples with reduced SPHK1&2 mRNA (lower than the two-thirds) are associated with increased month disease free rates and increased month overall survival rates (Supplementary Figures 1B and 1C), and a trend towards reduced mRNA expression levels of CCN1 and CCN2 (Supplementary Figures 1D and 1E). In light of emerging evidence for the critical role of S1P in the carcinogenesis of ovarian cancers, future studies aimed at targeting S1P signaling or its synthesis for therapeutic strategies will be of great interest.

Disruption of actin-polymerization is a critical process that cancer cells obtain enhanced motility and invasiveness during cancer cell progression [30]. Previous studies have shown that S1P can induce cytoskeleton reorganization, thereby regulate cell motility and invasiveness by regulating cytoskeleton dynamics [31, 32]. Furthermore, S1PRs and Rac/Rho have been reported to play fundamental roles in the regulation of S1P-induced cell migration [32]. In normal physiology, S1P could also promote ovarian follicle growth by enhancing its actin polymerization [33]. The actin cytoskeleton is known to be one of the upstream regulators of Hippo signaling to activate YAP activity and maintain its activating mode.[34] In the present study, we did not show how S1P interrupts the Hippo signaling. In particular, whether S1P can decrease YAP phosphorylation and increase YAP intranucleus activity through disrupting the actin-polymerization remain to be elucidated.

CCN family members are aberrantly expressed in various cancer tissues and are responsible for the promotion or inhibition of cancer progression in a cell-specific manner [35]. Using the Kaplan-Meier analysis of patients with serous ovarian cystadenomas from The Cancer Genome Atlas (TCGA) (n=607), we have demonstrated that samples with reduced CCN1/CCN2 mRNA levels are associated with increased month disease free rates and increased month overall survival rates (Supplementary Figures 2B and 2C). Consistent with these clinical data, our experimental results showed that knockdown of the endogenous CCN1 and CCN2 significantly decreased the cell proliferation in EOC cells, indicating that CCN1 and CCN2 might play a pivotal role in the progression and outcome of ovarian cancers. Furthermore, concomitant knockdown of CCN1 and CCN2 completely abolished the S1P-induced cell proliferation, indicating that both CCN1 and CCN2 mediate the downstream cellular action of S1P. Our study also showed that S1P promptly increased the expression of CCN1 and CCN2 as soon as 1 h after treatment, and the stimulated effect gradually decreased after reaching a peak at 2-3 h. However, the effect of S1P-induced cell proliferation may maintain up to 48 h. The reason why the transient up-regulation of CCN1/CCN2 can maintain a stimulatory effect on cell proliferation for a long period is probably because that the CCN family may mediate its function by binding to integrins (transmembrane receptors) or other membrane receptors [36]. Once CCN family members were secreted, they can bind to the integrin receptors and trigger downstream signaling and the subsequent genes expression. Future studies will focus on the interaction and connection of the CCN ligands and the membrane receptors.

In summary, we have demonstrated that the core components of Hippo signaling are expressed in EOC cells. In addition, our results indicate that S1P disrupts Hippo signaling by reducing YAP phosphorylation and increasing the expression of CCN1 and CCN2. Furthermore, the increase in CCN1/CCN2 expression contributes to the S1P-induced increase in cancer cell proliferation. Our *in vitro* results suggest that S1P and CCN1/CCN2 may play crucial roles in the development and progression of ovarian cancers.

## MATERIALS AND METHODS

### Cell culture

The OVCAR3 and SKOV3 human ovarian cancer cell lines were obtained from the American Type Culture Collection (Manassas, VA). Cells were grown in a 1:1 (v/v) mixture of M199/MCDB105 medium (Sigma–Aldrich, Oakville, ON) supplemented with 10% fetal bovine serum (FBS; Hyclone Laboratories Inc., Logan, UT), 100 U/ml of penicillin (Life Technologies, Inc/BRL, Grand Island, NY, USA), 100 μg/ml of streptomycin sulfate (Life Technologies), and maintained at 37°C in a humidified 5% CO_2_ atmosphere. The culture medium was changed every other day in all of the experiments.

### Antibodies and reagents

Monoclonal anti-YAP, monoclonal anti-cyclin D1, polyclonal anti-cleaved caspase 3 and polyclonal anti-pYAP antibodies were obtained from Cell Signalling Technology (Danvers, MA). Polyclonal anti-CCN1 (Cyr61), anti-CCN2 (CTGF) and monoclonal anti-GAPDH antibodies were obtained from Santa Cruz Biotechnology (Santa Cruz, CA). Horseradish peroxidase-conjugated goat anti-mouse IgG and goat anti-rabbit IgG were obtained from Bio-Rad Laboratories (Hercules, CA). S1P was obtained from Avanti Polar Lipids (Alabaster, AL). Verteporfin (VP) was obtained from VWR International (Randor, PA). For the VP experiments, cells were treated with 10 μm of VP for 10 min, washed and then treated with 300 nM of S1P for additional 2 h. Cycloheximide (#1041) was obtained from BioVision (Mountain View, CA).

### Immunofluorescence staining

OVCAR3 or SKOV3 cells were fixed with 4% paraformaldehyde in phosphate-buffered saline (PBS) for 15 min. Following permeabilization (in 0.1% Triton, 0.1% sodium citrate for 10 min) and blocking (in Dako blocking solution for 1 h), cells were incubated with YAP primary antibodies (1:100 dilution) overnight at 4°C. After washing with PBS, cells were incubated with fluorescent tag conjugated secondary antibodies, Alexa Fluor 488 (Invitrogen, 1:500 dilutions) for 30 min in the dark. Samples were mounted using ProLong Gold antifade reagent with DAPI (Invitrogen) for 5 min, and viewed under fluorescent microscopy, as previously described [37].

### Small interfering RNA (siRNA) transfection

We performed transient knockdown assays with an ON-TARGET*plus* non-targeting control pool or separate ON-TARGET*plus* SMARTpools targeting YAP, CCN1 or CCN2 (Thermo Fisher Scientific). The cells were pre-cultured to 50% confluence in antibiotic-free M199/MCDB105 medium containing 10% FBS and then transfected with 50 nM siRNA using Lipofectamine RNAiMAX (Life Technologies) for 24 h or 48 h, as previously described [38–41]. The knockdown efficiency for each target was confirmed using Western blot analysis.

### Reverse transcription quantitative real-time PCR (RT-qPCR)

Cells were washed with cold PBS, and total RNA was extracted with TRIzol Reagent (Life Technologies) according to the manufacturer's instructions. RNA (3 μg) was reverse transcribed into first-strand cDNA with random primers and Moloney Murine Leukemia Virus (MMLV) reverse transcriptase (Promega, Medison, WI, USA). RT-qPCR was performed on the Applied Biosystems 7300 Real-Time PCR System in 96-well optical reaction plates. Each 20 μL RT-qPCR reaction contained 1X SYBR Green PCR Master Mix (Applied Biosystems), 20 ng of cDNA and 250 nM of each specific primer. The specificity of each assay was validated using a dissociation curve analysis and agarose gel electrophoresis of the PCR products. The assay performance was validated by evaluating amplification efficiencies using means of calibration curves, and ensuring that the plot of the log input amount *vs*. the ΔCq (also known as ΔCt) had a slope < |0.1|. Three separate experiments were performed on different cultures and each sample was assayed in triplicate. Mean value was used for the determination of the mRNA levels using the comparative ΔCq (ΔCt) method with the formula 2^−ΔΔCq^ (2^−ΔΔCt^) and GAPDH as the reference gene.

### Western blot analysis

After treatment, the cells were washed with cold PBS and lysed in lysis buffer (Cell Signaling) containing protease inhibitor cocktail (Sigma-Aldrich). Extracts were centrifuged at 20,000 x *g* for 15 min at 4°C to remove cellular debris, and the protein concentrations were quantified using the DC Protein Assay (Bio-Rad Laboratories Inc.). Equal amounts of protein were separated using 10% SDS-PAGE and transferred to polyvinylidene fluoride membranes. The membranes were blocked for 1 h in Tris-buffered saline containing 0.05% Tween 20 and 5% nonfat dried milk, and then incubated overnight at 4°C with the relevant primary antibodies. After washing, the membranes were incubated with a peroxidase-conjugated secondary antibody (Bio-Rad) for 1 h. Immunoreactive bands were detected using enhanced chemiluminescence reagents or a SuperSignal West Femto Chemiluminescence Substrate (Pierce, Rockford, IL, USA), followed by exposure to CL-XPosure film (Thermo Fisher, Waltham, MA, USA). Membranes were stripped with stripping buffer (50 mM Tris-HCl pH 7.6, 10 mmol/l β-mercaptoethanol and 1% SDS) at 50°C for 30 min, and then reprobed with GAPDH antibody as a loading control.

### Proliferation assay

The cells were seeded in a 96-well plate for 24 h, and treated with S1P or PET solution (as a vehicle control) for an additional 72 h. The medium was changed every 48 h. After the incubation, 50 μf of MTS (5 mg/mL) was added to the medium, and the plates were incubated for an additional 2 h. The optical density was then measured at 490 nm using a microplate spectrophotometer (Dynex technologies, Sullyfield, VA).

### Statistical analysis

PRISM software (GraphPad Software, Inc., San Diego, CA, USA) was used to perform one-way ANOVA followed by Tukey's multiple comparison tests. The results are presented as the mean ± SEM of at least three separate experiments performed on different cultures, and were considered significantly different from each other if *P* < 0.05.

## SUPPLEMENTARY FIGURES


