# Supplementary material for: Sphingosine-1-phosphate promotes ovarian cancer cell proliferation by disrupting Hippo signaling
Source: Oncotarget. 2017 Feb 24;8(16):27166–76. doi: 10.18632/oncotarget.15677 (PMC5432326; doi:10.18632/oncotarget.15677)
Supplement: Supplementary file 1 [file oncotarget-08-27166-s001.pdf]

Sphingosine-1-phosphate promotes ovarian cancer cell proliferation by disrupting Hippo signaling

SUPPLEMENTARY FIGURES

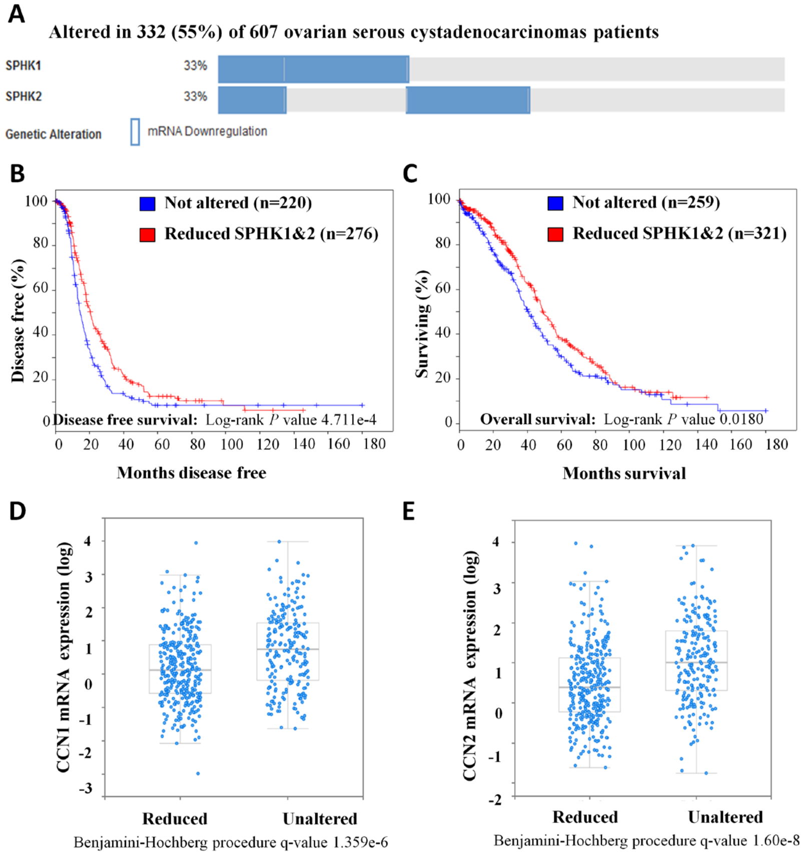

Supplementary Figure 1: Reduced SPHK1&2 is associated with elevated disease free survival and elevated overall survival in ovarian serous cystadenocarcinoma samples. **(A)** The cBioPortal for Cancer Genomics was used to query serous ovarian cystadenomas from The Cancer Genome Atlas (n=607) for down-regulation of SPHK1&2 mRNA lower than the two-thirds. OncoPrint shows cases with reduced mRNA levels of SPHK1 and SPHK2 across all 607 ovarian serous carcinomas. **(B-C)** Month disease free rate **(B)** and month overall survival rate **(C)** between unaltered samples and those with reduced SPHK1&2 are displayed as Kaplan-Meier survival curves with a *P* value from a Log-rank test. **(D-E)** Reduced SPHK1&2 expression contributes to the down-regulation of CCN1/CCN2 mRNA expression in serous ovarian cancers. Enrichment analysis comparing CCN1 mRNA and CCN2 mRNA levels between unaltered samples and those with SPHK1&2 mRNA levels in the lower 1/3 (reduced). Levels of CCN1 mRNA **(D)** or CCN2 mRNA **(E)** (from microarray) are displayed as box plots with a *Q* value using a Benjamini-Hochberg procedure.

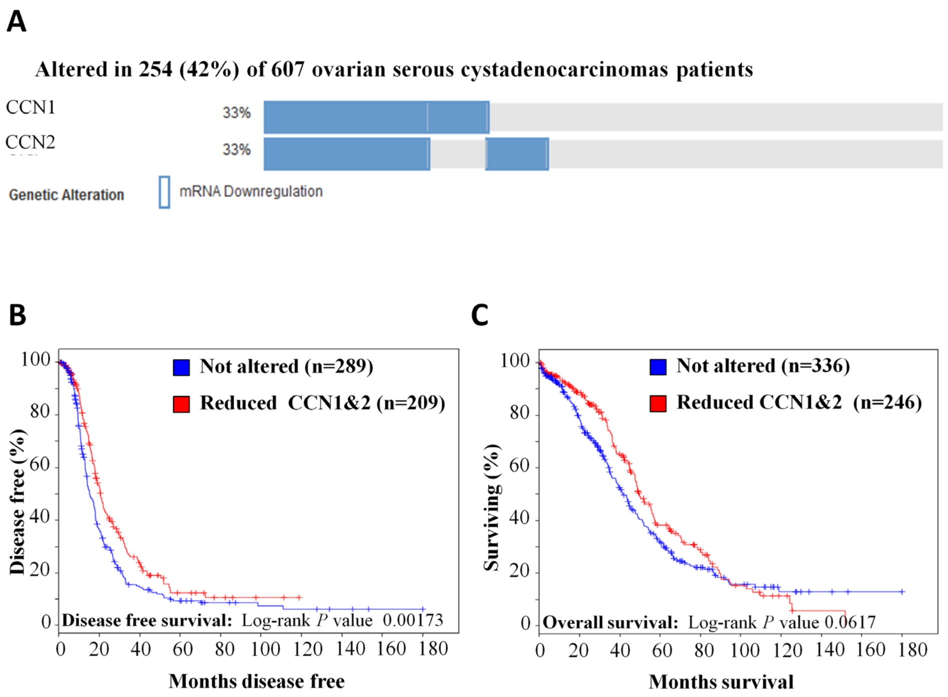

Supplementary Figure 2: Reduced CCN1/CCN2 expression is associated with elevated disease free survival rate and elevated overall survival rate in ovarian serous cystadenocarcinoma samples. **(A)** The cBioPortal for Cancer Genomics was used to query serous ovarian cystadenomas from The Cancer Genome Atlas (n=607) for down-regulation of CCN1/CCN2 mRNA lower than the two-thirds. OncoPrint shows cases with reduced mRNA levels of CCN1 and CCN2 across all of the 607 ovarian serous carcinomas. **(B-C)** Month disease free rate **(B)** and month overall survival rate **(C)** between unaltered samples and those with reduced CCN1/CCN2 are displayed as Kaplan-Meier survival curves with a *P* value using a Log-rank test.
